# Clinical symptoms and risk factors in people with mental disorders: findings from the interRAI emergency screener for psychiatry in Brazil

**DOI:** 10.3389/fpsyt.2026.1736120

**Published:** 2026-01-29

**Authors:** Crystalyn Rocho de Borba, Johanna de Almeida Mello, John P. Hirdes, Elton Luiz Ferlin, Alice Hirdes

**Affiliations:** 1 Graduate Program in Health Promotion, Lutheran University of Brazil, Canoas, Brazil; 2 Department of Oral Health Sciences, KU Leuven, Leuven, Belgium; 3 School of Public Health Sciences, University of Waterloo, Waterloo, ON, Canada; 4 Hospital de Clínicas de Porto Alegre, Biostatistic and Data Analysis Unit, Porto Alegre, Brazil; 5 Graduate Program in Nursing, Federal University of Rio Grande, Rio Grande, Brazil

**Keywords:** drug abuse, emergencies, general hospital, mental health disorders, risk factors, screening

## Abstract

**Background:**

Emergency rooms in general hospitals receive patients experiencing acute crises, exacerbations of chronic mental disorders, and psychiatric emergencies. This study aims to describe the main characteristics and clinical symptoms of the sample of mental health patients across main diagnoses, and to identify the risk factors of suicide, as well as of hetero-aggression. This study aims to investigate the associations between clinical symptoms, risk factors for suicide attempts and hetero aggression, and psychiatric diagnoses in patients with mental disorders and addictions.

**Method:**

This is a quantitative, cross-sectional, and analytical study. The Emergency Screener for Psychiatry instrument was applied to screen patients in an Emergency Care Unit in Primary Health Care and a Mental Health Unit of a University Hospital in Brazil. A comparative analysis of the main characteristics of patients between the sites was conducted. Logistic models were used to identify risk factors of suicide attempts and hetero aggression.

**Results:**

The scales of depression severity (OR:2.69), risk of harm to others (OR:3.31), and social withdrawal (OR:4.27) were identified as risk factors for suicide attempts. A protective factor was the item reporting if family/friends or professionals are concerned about the patient’s self-harm (OR:0.34). For hetero-aggression risk, using the harm to others scale, only the mania scale (OR:4.31) and history of four or more previous hospitalizations (OR:2.80) were significant. In both models, no significant associations were found for the type of diagnosis or the patient’s admission site.

**Conclusion:**

The Emergency Screener for Psychiatry proved to be a useful screening instrument to identify risk factors of suicide attempts and hetero aggression of patients in emergencies settings and general hospitals.

## Introduction

Data from the World Health Organization (WHO) indicate that in the global population, one in eight people live with mental disorders, regardless of social class, gender, ethnicity, culture, or country. Approximately one billion people suffer from psychological disorders, and after the pandemic, there was a 25% increase in the prevalence of depression and anxiety cases (1). Studies (2, 3) indicate that psychiatric emergency services are struggling with increased patient demand, leading to overcrowding in emergency units, lack of qualified professionals, and inadequate physical structures.

Psychiatric emergencies can include psychoses, agitation, anxiety, substance use, mood disorders, suicidal or homicidal behavior, as well as overlapping clinical conditions such as delirium and dementia (3). Emergency situations represent complications of mental disorders and may involve social exposure, restraints and more aggressive approaches (4).

Brazilian national studies (5–9) show that mental disorders are a crucial part of emergency care in general hospitals. The literature (5–12) indicates that the most prevalent mental disorders include schizophrenia, anxiety disorders, disorders due to the use of psychoactive substances, depressive and mood disorders, neurodevelopmental disorders, stress-related and somatoform disorders, and schizotypal and delusional disorders. Regarding suicide ideation or attempt, connecting individuals in need of psychiatric treatment with adequate medical services has proven to be an effective suicide prevention strategy in Japan (13).

Evaluating suicidal ideation in emergencies can alert professionals to subsequent suicidal acts and offer insights into the patient’s degree of psychological distress (14). However, despite attending to patients with mental disorders and suicide risk in emergencies, clinicians feel emotional distress and discomfort asking about suicidal ideation due to fear of feeling responsible, contributing to avoidant anxious behavior (15). Currently, with the increase in patients with psychiatric complaints arriving at emergencies, professionals need to have knowledge about conducting psychiatric interviews (16).

A retrospective cohort study conducted in England examining patient characteristics and healthcare resource utilization revealed that individuals with major depressive disorder (MDD) and moderate to high suicidal intent are generally younger, predominantly male, and present with higher rates of comorbid anxiety, substance abuse, and healthcare utilization compared to those without suicidal intent (17). These findings underscore the urgent need for more effective medical interventions to alleviate symptoms, reduce suicide-related mortality, and mitigate the burden on healthcare systems.

Suicide prevention strategies should also incorporate the assessment of physical aggression and impulsive behavior, which may act as proximal facilitators of suicidal acts. Notably, impulsivity scores among females were significantly higher in the low-lethality suicide attempt group than in suicide ideators, whereas levels of hostility and aggression were significantly elevated among females with high-lethality suicide attempts compared to ideators (18). A study (19) highlights that an initial presentation to the emergency department (ED) for self-harm is a significant risk factor for subsequent self-harm and suicide, showing that approximately one in four individuals later re-present to the ED with recurrent self-harm episodes.

A systematic review identified that self-harm and aggression are driven by their association with separate self-harm or aggressive behaviors. These findings suggest that dual harm does not constitute a distinct clinical entity. Rather, complex interactions between risk factors associated with self-harm and aggression, as well as their multiplicative effects, may lead to dual harm (20). In a general adult psychiatric setting, patients at risk for violent behavior, especially dual-harm patients, should be identified and monitored as part of the risk assessment (21).

People with mental disorders have the right to receive immediate and qualified assistance during psychiatric crises (3). Suicide attempts and hetero aggression are key clinical outcomes in emergency mental health care, as they represent critical *risk predictors* for adverse events and potential need for hospitalization. However, there is a lack of mental health assessment instruments in emergencies in Brazil that address mental state, social support, self-care ability, and risk of self-harm and hetero aggression.

In this context, the interRAI Emergency Screener for Psychiatry (ESP) (22) includes specific domains designed to identify these risks, particularly through the Severity of Self-Harm, Risk of Harm to Others, and Self-Care Index scales, which support early risk recognition and clinical decision-making across emergency settings. The inclusion of the Life Stress Events Scale (23) aimed to assess whether patients had experienced potentially stressful life events during the previous year. Such events may trigger crises, mental disorders, and increased risks for the patient.

Given these considerations, the study aims to describe the main characteristics and clinical symptoms of the sample of mental health patients across main diagnoses, and to identify the risk factors of suicide, as well as of hetero aggression.

## Method

### Study design, location, and participants

The present study has a quantitative, cross-sectional, and analytical design, guided by the Strengthening the Reporting of Observational Studies in Epidemiology (STROBE) tool. The research was conducted at a University Hospital and an Emergency Care Unit (UPA) in the Metropolitan Region of Porto Alegre in Rio Grande do Sul, Brazil. The mental health unit at the University Hospital (HU) has 20 beds for adult patients, with psychiatric hospitalizations lasting usually 21 to 30 days. The UPA has 2 isolation beds and 12 observation beds, divided between the emergency and urgency rooms. The interviews were carried out less than 24 hours after the patient entered the hospital, or the UPA, from March to December 2023.

The instrument was applied to 182 participants, with 80 interviews conducted in the Mental Health Unit of the University Hospital (HU) and 102 interviews with individuals requesting psychiatric evaluation at the Emergency Care Unit (UPA). All patients were interviewed within the first 24 hours of entering the services. If the patient could not respond to a question, a family member, if present, could respond.

### Sample definition

A sample size calculation was performed before the study using GPower software version 3.1. Considering an effect size of 0.5, 90% power, and a 0.05 α error probability, the minimum sample size for the analysis was 172, approximately 86 in each site.

### Selection criteria

Inclusion criteria were participants aged 18 years or older, of both sexes, with mental disorders, hospitalized in the Mental Health Unit for less than 24 hours; and patients awaiting psychiatric evaluation requested by professionals, usually general physicians, from the Emergency Care Unit (UPA) for less than 24 hours. Exclusion criteria were patients under 18 years of age, those requiring isolation (e.g., COVID-19), psychomotor agitation, or those with reduced level of consciousness, intoxication, or withdrawal from psychoactive substances.

### Instruments and variables of the study

#### InterRAI emergency screener for psychiatry

The first instrument used was the ESP, designed to evaluate patients with mental disorders in crisis. It has 13 sections with sub-items that indicate risks of self-harm, hetero-aggression, self-care, and the ability to care for others. ESP aims to assist in diagnosis and care planning focused on patient and staff safety. The ESP was developed by the interRAI network, a collaborative network of researchers and professionals in over 35 countries, committed to improving healthcare services for people with complex medical conditions, maintaining high scientific quality standards.

The pilot version of the instrument was tested in Canada, Finland, and Brazil. The ESP demonstrated statistical consistency and reliability, making it suitable for screening use in emergencies and mental health services, with its psychometric properties fitting the internal structure (23). The validation for the Brazilian context, the ESP showed adequate psychometric properties through internal structure analysis, evaluated by Cronbach’s Alpha and McDonald’s Omega indices, with values ranging from 0.60 to 0.94 (24).

Several scales from the interRAI ESP instrument were used in the analyses. The Aggressive Behavior Scale ranges from 0 to 12, with a cutoff value of >=7, the Depression Severity Index (0–15) has a cutoff >=8, the Mania Scale (0–20) has a cutoff >=9, and the Positive Symptoms Scale (0–12) has a cutoff >=6. The Risk of Harm to Others Scale (0–6) has a cutoff >=3, as does the Self-Care Index and the Severity of Self-Harm Scale. Finally, the Social Withdrawal Scale ranges from 0 to 12, with a cutoff of >=9. The scales are described in the interRAI Emergency Screener for Psychiatry (ESP) manual (22) and have been previously published (25). The ESP software automatically generates a risk summary based on the complete assessment, which includes four items: risk to oneself, risk to others, inability to care for oneself, and inability to care for dependents.

#### Life stress events scale

The second instrument used was the EVPE, which assesses stressors and factors that may impact health and well-being, covering areas such as health conditions, personal circumstances, financial issues, and experiences of violence. The construct validity of this instrument was confirmed in a study using a list of stressors known as the Life Stress Events Inventory. The EVPE provided evidence supporting the construct validity of stressful life events in two Brazilian samples and demonstrated good model fit in both samples (RMSEA < 0.05; CFI/TLI > 0.90). Using the EVPE allows investigating and quantifying the occurrence of stressors in a person’s life, providing valuable information to understand the impacts of these events on their health and well-being (23). The EVPE items were scored using dichotomous response categories (‘yes’/‘no’).

### Data collection, recruitment, and storage

The data collection was conducted by the first author of the research and a medical student intern. Both were trained in using the instrument and the software by the advisor. To support the application of the ESP, the instrument manual was used. Patients admitted in the last 24 hours to the Mental Health Unit of the University Hospital and the UPA were invited to participate, and after signing the Free and Informed Consent Form, the data collection was carried out. When necessary, information was confirmed by family members or the attending physician. Interviews were conducted sequentially across shifts, but not sequentially across the days of the week, according to the schedule made available by the institutions for data collection.

Data collection used dedicated software with internationally used interRAI coding, containing both the ESP and EVPE instruments. Initially, the ESP was applied, followed by the EVPE. The interviews took place in a specific room at the HU and available spaces at the UPA, with a medium time of 50 minutes to complete the ESP.

### Data analysis

First descriptive analyses were performed to describe patients’ sociodemographic data and differences across settings. In a second step, the most prevalent psychiatric diagnoses were described, as well as the prevalence of problems indicated by the ESP scales, the prevalence of suicide attempts, and hetero aggression. Independent t-tests and chi-squared tests were used for the calculation of the differences between the patients’ characteristics across settings and gender (26). ANOVA was used to explore whether characteristics and determinants varied according to psychiatric diagnoses.

The prevalence of life events was described across diagnoses and, finally, to investigate the associations between clinical symptoms and risk factors in patients with mental and/or addiction disorders.

After verifying that collinearity assumptions were met (based on Variance Inflation Factor VIF value<5), logistic regression models were built to assess the risk of suicide and hetero-aggression. These models were adjusted for potential confounders. The risk determinants that resulted in significant associations showed the factors that contribute most to these events (27). As the analyses were exploratory and intended to describe subgroup trends rather than infer definitive associations, multiple p-values were reported. All analyses were performed using STATA18.

### Ethical aspects

The project was developed following the guidelines and regulatory standards for research involving human beings and was approved under CAAE no. 61491922.6.0000.5349. All participants signed the Free Informed Consent Form. Ethical aspects regarding research with human beings were respected as determined by Resolution No. 466/2012 (28).

## Results

A total of 182 patients were interviewed, with ten interviews excluded from the sample due to missing data on the patient’s age or other socio-demographic information. Due to missingness in diagnostic information, 16 patients were excluded in the second step of the analysis.

**Table 1** shows data from 172 patients attended at the UPA and HU within the first 24 hours of symptom onset. The total sample consisted of 56.98% men with a mean age of 41.5 (± 14.7) years. Most patients were admitted at the family’s request (60.1%), and 38.95% had a history of 1 to 3 previous admissions. Psychiatric hospitalization in Brazil is divide in three modalities based on the degree of patient consent and the authority responsible for the decision to hospitalize: voluntary hospitalization, conducted with the patient’s free and informed consent; involuntary hospitalization, which occurs without the patient’s consent and at the request of a third party, typically a family member or legal guardian; and hospitalization by judicial determination (compulsory hospitalization), which is ordered by a court.

**Table 1:** Descriptive characteristics of the samples.

Characteristics	HU n=77	UPA n=95	Total sample n=172	P-values between HU and UPA
Age (± SD)^†^	40.29 (± 14.88)	42.51 (± 14.58)	41.51 (± 14.71)	0.456
Sex male (%)	61.04%	53.68%	56.98%	0.333
The person has dependents	37.98%	34.74%	36.63%	0.567
Most common referral methods				
The same person	21.43%	23.08%	22.56%	0.000***
Family or acquaintances	47.62%	65.93%	60.15%	0.000***
Secondary care professional (CAPS)	23.81%	1.10%	8.27%	0.000***
Judicial order or police	7.14%	9.89%	9.02%	0.000***
Number of hospitalizations				
0 (none)	18.18%	25.26%	22.09%	0.381
1–3	41.56%	36.84%	38.95%	
4–5	23.38%	15.79%	19.19%	
6 or more	16.88%	22.11%	19.77%	

^†^SD, Standard deviation of the mean. ***p<0.001.

A total of 77 participants were interviewed at the HU, of which 61.04% were men. The most frequent referral forms to the HU were 47.62% by the family network, 23.81% by the Psychosocial Care Center (CAPS), and 21.43% of patients requested their own admission. The UPA sample included 95 participants, of which 53.68% were men, with the most frequent referral forms by the family network (65.93%) or the patients themselves requesting admission (23.08%). No significant differences were found between locations for age, sex, and having dependents. The only significant differences between the samples were found for the form of referral.

**Table 2** shows the main diagnoses in the sample and their clinical characteristics, as well as the ESP instrument scales. The most prevalent mental health disorders in the sample are addictions (32.56%) and schizophrenia (26.74%). Significant differences were found indicating a higher presence of patients with schizophrenia and anxiety disorders in the hospital, while more depressive disorders were present in the UPA. In the sample the diagnoses were clinical, made by psychiatrists, using the DSM5.

**Table 2:** Clinical characteristics of the sample.

Characteristics	Addictions n=56	Anxiety n=22	Depression n=46	Schizophrenia n=32	P-values*
Has had hospitalizations	92.86%	54.55%	71.74%	90.63%	0.000***
Several hospitalizations (4 or more)	44.64%	18.18%	39.13%	56.25%	0.044*
Positive Symptoms Scale	7.14%	9.09%	41.30%	94.38%	0.000***
Family members concerned about self-harm	35.71%	86.36%	28.26%	59.38%	0.000***
Inability to care for dependents	50.00%	27.27%	60.87%	75.00%	0.004**
Mania Scale	50.00%	40.91%	36.96%	65.63%	0.000***
Aggressive Behavior Scale	18.87%	4.55%	16.67%	37.04%	0.034*
Depressive Severity Index	41.07%	77.27%	73.91%	34.38%	0.000***
Mild to severe cognitive impairment.	25.00%	4.55%	30.43%	68.75%	0.000***
Hygiene Problems	10.71%	4.55%	4.35%	40.63%	0.000***
Social Withdrawal Scale	85.71%	95.45%	80.43%	53.13%	0.000***
Risk of Harm to Others Scale	53.57%	63.64%	54.35%	75.00%	0.197
Severity of Self-harm Scale	35.71%	54.55%	73.91%	78.13%	0.000***
Suicide Plan last 30 days	14.29%	31.82%	60.87%	9.38%	0.000***
Suicide attempt	41.07%	40.91%	65.22%	34.38%	0.026*

*p<0.05, **p<0.01, ***p<0.001.

Regarding the clinical characteristics of the total sample, 75.58% of patients had a high degree of social withdrawal according to the social withdrawal scale, with similar percentages in both samples. The risk of harm to others scale showed that 62.21% presented risks of hetero aggression, and the self-harm severity scale showed that 59.88% of patients had a risk of severe self-harm, both scales without significant differences between samples.

**Table 3** shows that interviewees with significantly higher rates of financial difficulties were patients with addiction (80.36%) followed by patients with anxiety diagnosis (72.73%). Addicted patients were also the most forced to move residence (50.0%), victims of aggression (39.29%), or robbery (12.5%).

**Table 3:** Life stress events scale.

Characteristics	Addictions n=56	Anxiety n=22	Depression n=46	Schizophrenia n=32	P-values*
Hospitalization	12.50%	18.18%	8.89%	15.63%	0.703
Death of family member	8.93%	0%	17.78%	6.25%	0.102
Financial difficulties	80.36%	72.73%	48.89%	37.50%	0.000***
Forced to move from home	50.0%	18.18%	11.11%	21.88%	0.000***
Romantic breakup	28.57%	36.36%	22.22%	9.38%	0.096
Robbed	12.50%	0%	0%	3.13%	0.018*
Victim of assault	39.29%	4.55%	13.33%	12.50%	0.001**
Health issues	21.43%	18.18%	4.44%	12.50%	0.099

*p<0.05 **p<0.01 ***p<0.001.

The EVPE instrument analysis showed that 60.23% of interviewees had financial difficulties, 27.49% were forced to move, 22.81% had a marital breakup, 22.22% suffered aggression, 16.96% faced a health problem in the last year, 15.79% were hospitalized, 11.70% were mourning a family member’s death in the past twelve months, and 5.26% were robbed. Data analysis from the two institutions showed similar results, with the most significant being financial difficulty for both populations. At the UPA, 18.09% were affected by a family member’s death, while only 3.90% were affected at the HU.

The data showed that women (41.89%) planned to commit suicide significantly more than men (18.37%, p=0.001), and suicide attempts were significantly more frequent among women (54.05%) than men (36.73%, p=0.024). The logistic regression model in **Table 4** shows the risk factors for suicide attempts. The following scales showed significant odds ratios: depression severity scale (2.69), risk of harm to others scale (3.31), and social withdrawal scale (4.27). A protective factor found in the item Family/friend or professionals’ concern about self-harm damage showed an odds ratio below 1, being 0.34, indicating that the suicide risk for these patients is significantly lower. No significant associations were found between suicide attempts and diagnoses or the patient’s admission location.

**Table 4:** Logistic model for suicide attempts.

Factors	Beta coefficient	Suicide attempts pseudo R2:0.2562 C-statistic: 0.8067 α= −2.59 (CI: -4.73; -0.45)			
	β	O.R.^§^	CI−^†^	CI+^‡^	P-value
Age ≥50	−0.56	0.57	0.22	1.44	0.235
Sex (ref: female)	−0.39	0.67	0.27	1.68	0.394
Previous hospitalizations	1.02	2.77	0.90	8.51	0.076
Diagnoses (ref: Addiction)					
1- Anxiety disorder	0.26	1.30	0.31	5.38	0.720
2- Depressive disorder	0.89	2.43	0.67	8.85	0.177
3- Schizophrenia	0.90	2.45	0.57	10.55	0.229
Mania scale (>=9)	−0.90	0.41	0.15	1.11	0.078
Depression Severity Scale	0.99	2.69	1.06	6.82	0.036*
Positive Symptoms Scale (>=6)	−0.71	0.49	0.16	1.51	0.213
Risk of Harm to Others Scale	1.20	3.31	1.30	8.57	0.013*
Social Withdrawal Scale	1.45	4.27	1.27	14.33	0.019*
Family members concerned about self-harm	−1.08	0.34	0.14	0.85	0.02*
Stress factor	−0.08	0.92	0.40	2.13	0.853
UPA (ref: HU)	0.46	1.59	0.63	3.99	0.324

**^§^**O.R.: Odds ratios. ^†^CI−: Lower bound Confidence Interval 95%. **^‡^**CI+: Upper bound Confidence Interval 95%. *p<0.05.

The second logistic regression model on **Table 5** shows the risk factors for hetero aggression, using the harm to others scales. Only the mania scale and the history of multiple hospitalizations (4 or more) were significant factors, with the following odds ratios: 4.31 for the mania scale and 2.80 for previous hospitalizations. No other significant factors were found, and there were no significant associations between hetero-aggression and diagnoses or the patient’s admission location.

**Table 5:** Logistic model for hetero aggression.

Factors	Beta coefficient	Hetero-aggression pseudo R2: 0.2034 C-statistic: 0.7858 α= −0.37 (CI: −1.50; 2.24)			
	β	O.R.^§^	CI−^†^	CI+^‡^	P-value
Age ≥50	−0.35	0.70	0.28	1.78	0.458
Sex (ref: female)	−0.18	0.84	0.33	2.10	0.704
Previous hospitalizations	1.03	2.80	1.25	6.29	0.013*
Diagnoses (ref: Addiction)					
1- Anxiety disorder	0.79	2.20	0.59	8.13	0.238
2- Depressive disorder	−0.04	0.96	0.28	3.32	0.951
3- Schizophrenia	0.04	1.05	0.24	4.54	0.952
Mania Scale (>=9)	1.46	4.31	1.68	11.03	0.002**
Depression Severity Scale	−0.02	0.98	0.38	2.51	0.963
Positive Symptoms Scale (>=6)	0.72	2.06	0.72	5.95	0.180
Social Withdrawal Scale	−0.79	0.45	0.13	1.60	0.219
Stress factor	−0.53	0.59	0.25	1.38	0.222
UPA (ref: HU)	−0.18	0.83	0.32	2.11	0.690

**^§^**O.R.: Odds ratios. ^†^CI−: Lower bound Confidence Interval 95%. **^‡^**CI+: Upper bound Confidence Interval 95%. *p<0.05, **p<0.01.

## Discussion

This study investigated the main characteristics and clinical symptoms of the sample of hospitalized mental health patients across main diagnoses, and to identify the risk factors of suicide, as well as of hetero-aggression. The study participants were predominantly male, with an average age of 41 years. The study population is similar to other national and international studies, where the main disorders identified in emergency and urgency are depressive, anxiety, and schizophrenia disorders (8, 9, 11, 29, 30). Predictors of hospitalization include a history of psychiatric hospitalization, suicidal thoughts, and a mood disorder or schizophrenia/schizotypal/delusional disorder diagnosis (12).

National studies (5, 31) refer to the main disorders evidenced in emergencies as mood disorders, psychotic disorders, and disorders due to psychoactive substance use. Suicide attempts also have high rates in emergency care in Brazil, as well as self-inflicted violence, behaviors more associated with females (32, 33). As already evidenced in the literature, our results corroborate previous research (32–34), where suicide attempts were more associated with depressive disorders and females.

The greater susceptibility of females to self-harm behavior is related to high rates of domestic violence, sexual violence, moral violence, financial vulnerability, and cultural aspects related to gender inequality (35). However, males have the highest mortality rates, being more common in the population aged 60 years or older (36, 37). Deaths related to males are associated with violent suicide attempts such as hanging, jumping from heights, and traffic accidents (38).

Our results are analogous to other studies (30, 39–41) that refer to depressive disorders, self-harm behavior, financial difficulties, violence, and alcohol or tobacco use associated with a higher prevalence of suicide attempts. A cohort study (42) with patients with Major Depressive Disorder (MDD) and suicidal behavior using DSM-5 identified that younger patients and those more likely to have comorbid psychiatric conditions such as personality disorders, substance use, and anxiety had a higher risk of suicide. The most important risk factors associated with suicidal behavior within 1 year after the onset of an MDD episode were a history of suicidal behavior and age, a history of substance use, sleep disorders, and the context of care in which the MDD was diagnosed using the ICD-10. Patients with depression should be assessed for self-harm risk, hetero-aggression, and the ability to care for themselves (41).

Suicide risk is also evidenced in patients with Bipolar Disorder (BD). Completed suicide is a major cause of death in bipolar disorder (BD) patients. Some factors related to completed suicide including early onset of illness, family history of suicide, previous attempted suicides, comorbid conditions and treatment (43). BD increases morbidity and mortality not only due to the disorder and associated suicide risk but also due to associated diseases and the side effects of medications used for treatment. All patients with BD need to be evaluated for suicidal ideation using a specific instrument that assesses this risk (44).

Research (45) showed the association between MDD and anxiety symptoms and identified that suicide attempts were associated with anxiety symptoms in young adult MDD patients, highlighting the importance of reducing anxiety symptoms in this population to prevent suicides. Climate changes that affect the most vulnerable populations also impact mental health and are related to anxiety disorders, eco-anxiety, depression, suicide, substance use, and behavioral problems (46).

Studies (47–49) with addicted patients showed a prevalence of males in situations of severe social vulnerability, corroborated in this research. Individuals who have experienced violence also present risk factors for psychoactive substance use (50). Alcoholism and drug use are referred to as risk factors for depressive disorders (51–53). Multiple diagnoses, such as substance abuse and mental disorders, are frequently reported in emergency patients with suicidal ideation (54).

Our research presents similar results to international studies, with men showing more hospitalizations for schizoaffective disorders compared to women (55, 56). A third of patients with schizophrenia are also addicted, with the most used substances being cannabis, followed by cocaine and alcohol (57). Another study shows that 37.1% of patients diagnosed with schizophrenia were smokers, and 17.3% had harmful alcohol use (56). Schizophrenia patients also have suicide risks, with suicidal behavior, suicide attempts, and self-mutilation also evident in this population (58).

In Brazil, the Ministry of Health instituted a regulation in 2011 that makes it mandatory to report self-inflicted injuries and suicide attempts for all health services (18). Later, in 2014, a new regulation (59) required health secretaries to report suicide attempts within 24 hours, and in 2019, a law was approved (60) establishing the National Policy for the Prevention of Self-Mutilation and Suicide. However, despite the new regulations and law, national research (2) indicates that emergency and urgent care services are overcrowded, lacking physical infrastructure and specialized professionals to provide qualified care to patients in psychiatric crises.

Patients with suicidal ideation or behavior were often referred to emergency care within a short period (18% in less than a month). Those previously referred for ideation/plans had a 66% higher risk of transitioning to a suicide attempt, with 25% making this transition within a month after the previous referral (61). National cohort research (62) showed that patients with a current suicide attempt and a previous attempt in the last 6 months have an especially high risk of dying by suicide shortly after going to the emergency room.

Suicidal behavior causes functional losses, representing up to a third of the total years lived with disabilities, with high mortality rates among young people (43, 63). Suicidal ideation is closely associated with both suicide attempts and deaths, serving as a significant risk factor for future suicide attempts (14). Evidence suggests that a third of high-risk patients make new suicide attempts a month after being seen in emergencies (64).

From this perspective, patients discharged need follow-up in external mental health services (65). Therefore, risk screening and classification in emergencies for patients exhibiting suicidal or self-harm behavior should be conducted, along with the treatment of all individuals (3, 65). Thus, identifying suicidal ideation and early intervention reduce suicide rates (14, 66).

Our study showed the importance of family involvement in the prevention of self-harm. This finding is supported by recent literature (67) showing that family members play an important role in suicide prevention efforts, which highlights the need of strategies to empower families and to keep them involved in the care for the patient. The study also emphasized the need for prioritizing emotional and expressive support in prevention and treatment, such as therapy, peer networks, and community resources should focus here. Families also played a key role in keeping patients safe by limiting access to means of self-harm and providing valuable contextual information to healthcare professionals. To enhance their involvement, better communication and dissemination of safety and care plans, shared learning opportunities and tailored support for carers may be essential strategies (68).

## Conclusion

This study evidenced, using the ESP, that people with anxiety and depressive disorders present high rates of social withdrawal, risk factors for self-harm, suicidal ideation, and suicide attempts. Patients with schizophrenia had higher rates of rehospitalization, aggressive behavior, self-aggression, and hetero aggression. Schizophrenia patients also exhibit more positive symptoms and cognitive impairment. The mania and depression scales also indicated an increased risk of hetero and self-aggression, respectively. The self-harm scale showed that people with depression and schizophrenia have a higher suicide risk, while suicide plans and attempts are associated with depression. A protective factor for self-harm risk is the presence of family, friends, or professionals. The EVPE instrument demonstrated that patients with addictions report greater social vulnerability, are forced to move, and suffer violence.

The ESP proved to be a feasible instrument for use in emergency services and general hospitals, UPAs, and specialized services such as CAPS. At the end of the evaluation, the software generates a risk summary in four items: risk to oneself, risk to others, inability to care for oneself and dependents. These risks come from the scales Severity of Self-Harm (SoS), Risk of Harm to Others (RHO), and Self-Care Index (SCI). Identifying these risks can qualify care aimed at developing a personalized care plan. Additionally, a new instrument is being presented for use by different professional categories and in various contexts, such as emergency settings, mental health units in general hospitals, UPAs, and CAPS. The interRAI ESP is also useful for professional training at both undergraduate and postgraduate levels.

## Strengths, limitations, and future research

This study showed that the interRAI ESP is a feasible tool for psychiatric assessment in emergency settings, supporting decision-making where specialists in psychiatry are scarce. The tool can be a first help to guide care and can inform follow-up in integrated health systems. Limitations include missing sociodemographic data and a cross-sectional design, limiting causal conclusions. Despite the use of a non-representative sample, the findings provide valuable insights into a population that remains under-studied in emergency care settings. The minimum required sample size for the analysis was not achieved, and 16 patients were excluded due to missing diagnostic information; however, their inclusion is unlikely to have affected the results. A limitation of the research concerns working with the patient’s primary diagnosis. We suggest the use of secondary diagnoses in future research. A potential bias relates to the fact that all study participants were receiving care through public health services, which generally means they have lower income levels.

## Data Availability

The datasets presented in this article are not readily available because they were used under license for the current study, and are not publicly available. Requests to access the datasets should be directed to the corresponding author.
